# Antigenic Properties of N Protein of Hantavirus

**DOI:** 10.3390/v6083097

**Published:** 2014-08-13

**Authors:** Kumiko Yoshimatsu, Jiro Arikawa

**Affiliations:** Department of Microbiology, Graduate School of Medicine, Hokkaido University, Kita-ku, Kita-15, Nishi-7, Sapporo 060-8638, Japan; E-Mail: yosimatu@med.hokudai.ac.jp

**Keywords:** nucleocapsid, monoclonal antibody, epitope

## Abstract

*Hantavirus* causes two important rodent-borne viral zoonoses, hemorrhagic fever with renal syndrome (HFRS) in Eurasia and hantavirus pulmonary syndrome (HPS) in North and South America. Twenty-four species that represent sero- and genotypes have been registered within the genus *Hantavirus* by the International Committee on Taxonomy of Viruses (ICTV). Among the viral proteins, nucleocapsid (N) protein possesses an immunodominant antigen. The antigenicitiy of N protein is conserved compared with that of envelope glycoproteins. Therefore, N protein has been used for serological diagnoses and seroepidemiological studies. An understanding of the antigenic properties of N protein is important for the interpretation of results from serological tests using N antigen. N protein consists of about 430 amino acids and possesses various epitopes. The N-terminal quarter of N protein bears linear and immunodominant epitopes. However, a serotype-specific and multimerization-dependent antigenic site was found in the C-terminal half of N protein. In this paper, the structure, function, and antigenicity of N protein are reviewed.

## 1. Introduction

Species of the genus *Hantavirus* are classified in the family *Bunyaviridae.* Rodents, shrews, and bats are persistently infected with hantaviruses, whereas humans are infected accidentally, occasionally causing severe illness. Among rodent-borne hantaviruses, the causative agents of two important zoonoses are known: hemorrhagic fever with renal syndrome (HFRS) and *Hantavirus* pulmonary syndrome (HPS) [1,2]. 

Like other viral members of the *Bunyaviridae* family, hantaviruses are enveloped RNA viruses that contain three-segmented negative-sense RNAs, designated S, M, and L based on the molecular weight of their virion. The S, M, and L RNA segments encode nucleocapsid protein (N), envelope glycoproteins (Gn and Gc), and RNA-dependent RNA polymerase (L) protein, respectively [3]. Nonstructural (NS) protein was detected in the S genome segment of some hantaviruses as a candidate interferon antagonist [4,5,6]. Among the structural proteins, N protein is the most abundant in the hantavirus virion and accumulates in the cytoplasm of infected cells. Since N protein is immunodominant, diverse recombinant N proteins produced by various expression systems have been applied as diagnostic antigens to detect hantavirus-specific antibody (US patent number: 5614193) [7,8,9,10].

In addition to its value as a diagnostic antigen, N protein exhibits RNA-binding activity [11,12,13] and multimerization [14], which are crucial functions for the encapsidation of the viral genome in the virion [15]. Furthermore, N protein has been suggested to play an important role in the initiation of transcription and translation of the hantavirus genome. Thus, it is a multifunctional protein that contributes to not only virus encapsulation and assembly but also to the translation and transcription of genomic RNA to complete the viral lifecycle. 

Each species of *Hantavirus* appears to have a single predominant rodent, shrew, mole, or bat species that serves as its natural reservoir [16,17,18,19,20], probably because of the co-evolution of hantaviruses with their animal reservoirs. Rodent-borne hantaviruses comprise three large groups according to their host rodents: Murinae-, Arvicolinae-, and Sigmodontinae/Neotominae-associated. Due to the close association between rodent species and hantaviruses, their geographical distributions are the same. Therefore, Murinae- and Arvicolinae-associated hantaviruses, which are distributed in Eurasia, are called Old World hantaviruses. Alternatively, Sigmodontinae/Neotominae-associated hantaviruses are distributed in North and South American countries and are called New World hantaviruses [21]. Causative agents of HFRS and HPS are exclusive to rodents. Murinae-associated hantaviruses include causative agents of HFRS: Hantaan virus (HTNV), Seoul virus (SEOV), and Dobrava virus (DOBV). Thailand virus (THAIV), which is carried by *Bandicota indica,* is suspected of being pathogenic to humans in Asia [22]. Arvicolinae-associated hantaviruses are distributed throughout Eurasia, and Puumala virus (PUUV) is a causative agent of HFRS that has been called nephropathia epidemica (NE) in Northern Europe. Numerous Arvicorinae rodent-borne hantaviruses have been reported from both the Old World and New World. Among them, only PUUV is known to be pathogenic. The group of Sigmodontinae/Neotominae-associated hantaviruses, Sin Nombre virus (SNV), Andes virus (ANDV), Laguna Negra virus (LANV), and variable HPS-related hantaviruses were found in North and South America [23,24]. Hantavirus N protein shares a common antigenic site with each group.

Here, we review the antigenic properties of epitopes on hantavirus N protein, particularly in relation to the structure, virus species and function.

## 2. Hantavirus N Protein

### 2.1. Antigenic Profiling of N Protein Using Monoclonal Antibodies (MAbs) and Polyclonal Antibodies

The initial research using MAbs against N protein of HTNV, SEOV, and PUUV was reported by Ruo and coauthors [25]. Most clones against HTNV and SEOV were cross-reactive against two viruses but not cross-reactive with PUUV. Also, one serotype-specific clone against HTNV was established. Yoshimatsu *et al.* confirmed the binding region of MAbs produced by Ruo *et al.* All clones excluding the serotype-specific form were found to bind with the N-terminal part of N [26]. On the other hand, Lundkvist and coauthors reported MAbs against PUUV [27]. All clones against PUUV exhibited unique epitopes distinct from HTNV and SEOV. Among eleven clones, six were PUUV‑specific. The clone 3H9 was a PUUV-specific antibody and its epitope was determined as amino acids (aa) 251 to 260 (VKPGTPAQEI) using the pepscan assay [28]. However, epitopes of other MAbs were not determined with the same assay [28]. Elgh *et al.* showed that most PUUV‑specific MAbs bind to 100 aa of the N terminus of PUUV N [29]. MAbs against N protein of New World hantaviruses and against ANDV and Carizale virus (CARV) [30,31] were reported. In those studies, group-common, genus-common, and serotype-specific epitopes were found within the N-terminal region of N protein. MAbs against the N-terminal 120 aa of N of PUUV showed cross‑reactivity against New World hantaviruses, and these antibodies were useful for detecting antigens in immunohistochemical assays [32]. These results obtained by using MAbs indicate that the N terminus of hantavirus N protein is immunodominant.

Thottapalayam virus (TPMV) is a prototype virus of shrew-borne hantavirus and is the most distinct form of rodent-borne hantaviruses [33]. Shigel *et al.* reported MAbs against TPMV [34]. All four clones were directed against the N-terminal region. These results indicate that, even in TPMV, the N terminus of N protein is immunodominant. Furthermore, the N-terminal region showed strong reactivity in a Western blot assay on staining by MAbs and polyclonal antisera. This indicated that the epitope found in the N terminus is linear and immundominant [34]. Similarly, antibody epitopes induced in HFRS and HPS patient sera and rodent-borne hantavirus-infected animals using a synthetic peptide antigen and/or truncated antigens were investigated. The results indicated that linear and immunodominant epitopes of N protein are also present in the N-terminal region even in these polyclonal antibodies [28,34,35,36,37,38,39]. Based on the results of epitope-mapping studies with MAb observations, MAbs against N protein were mostly produced against the N-terminal region of N protein of Old World, New World, and shrew-borne hantaviruses. These results reveal that the basic structure of N protein may be common among viruses within the genus *Hantavirus*.

### 2.2. Deduced Structure of the N-Terminal Region of N Protein

**Figure 1:**
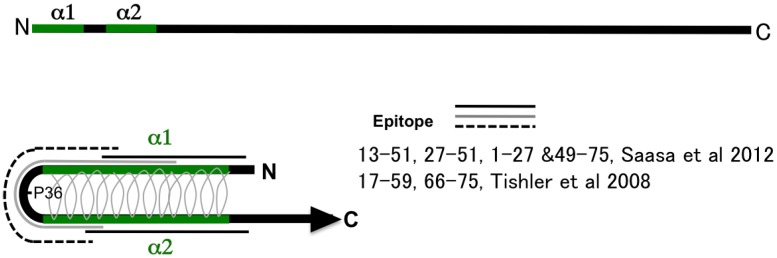
Schema of the structure of the N terminus of hantavirus N and epitope mapping. Two α helices α1 and α2, of the N-terminal region are shown [42]. The model of the intramolecular antiparallel coiled-coil structure has gained much support. Regarding the structure, several epitopes were reported as genus-common, type-common, or a type‑specific epitopes [30,31]. The turn of this structure was proline at the 36^th^ position.

As shown in Figure 1, secondary structure prediction based on the deduced aa sequence, two α-helices in the N-terminal region (α1 and α2), was reported by Alfadhli *et al.* [40,41]. At present, the intramolecular coiled-coil structure is a model of the N-terminal region favored by several researchers [42,43]. A coiled-coil structure of aa 1 to 74 based on crystal structure analysis [42] and NMR [44] was reported. Therefore, aa 1-74 produced two long helices (α1 and α2) that intertwine into a coiled-coil domain [44]. A schema of 100 aa of the N terminus of N protein is presented in the figure. The aa 1-74 produced the antiparallel coiled-coil structure of α1 and α2 helices shown in Figure 1A. The conserved proline at the 36th position was a vertex of the structure [42,44]. Based on the model, Tischler and Saasa proposed an antigenic model of the N-terminal region [30,31]. They found several overlapping epitopes in the N-terminal region by employing a competitive binding assay of MAbs. These MAbs showed various antigen-binding profiles including group-common, genus-common, and serotype-specific profiles. However, the N-terminal region seems to have only group-common epitopes and not to have serotype-specific or genus-common epitopes in actual infection. The N-terminal 100 amino acid antigens showed a group-common binding profile and low cross-reactivities with patient sera. Sera from HFRS patients infected with PUUV showed extremely low or no cross-reactivities against HTNV and DOBV antigens and slight cross-reactivity against SNV antigen [38,39]. The N-terminal part of N of ANDV and PUUV showed low cross-reactivities between Sigmodontinae/Neotominae-borne and Microtinae-borne hantaviruses [45]. These observations indicate that the antigenic region shown with black and solid lines (1-27 & 49-75) mainly induced antibodies in patient and animal sera during actual infection. The mutant N protein lacking aa 1-35 (α1-deletion mutant) completely lost its antigenicity [26]. Using MAb binding to the N-terminal 100 aa of HTNV N protein (such as GBO4, ECO2, and ECO1), it was not possible to determine their epitopes by a peptide-scanning assay employing a previously described peptide‑scanning assay [46]. These results also indicate that the coiled-coil structure of the N terminus was the main structure involved in the antigenicity of N. 

### 2.3. Deduced Structure of the Central Part of N Protein

**Figure 2:**
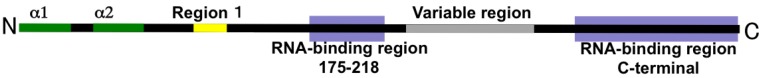
Schema of the central region of hantavirus N. Region 1 (aa 100-12) assists in N‑N homotypic interaction [49]. RNA-binding regions involving aa 175-218 [12] and 100 aa of the C terminus [11] are shown.

As shown in Figure 2, another report also showed that the central region of N protein was responsible for RNA-binding activity [12,13]. N protein may bind to hantaviral genomic RNA selectively rather than to general RNA; however, this has yet to be confirmed [11,47,48]. Yoshimatsu and coauthors reported that Region I (aa 100-125) was highly conserved among hantaviruses, and they identified an important region assisting in N-N homotypic interaction [49]. A variable region of N (aa 230-302) was identified in the central part of N protein (175-218) [50,51]. This region may be involved in the serotype-specific epitope of N protein.

### 2.4. Deduced Structure of the C-Terminal Region of N Protein

**Figure 3:**
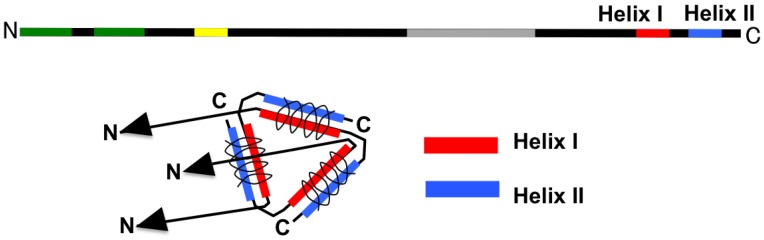
Interaction of the hantavirus N by using the C-terminal part. The coiled coil of Helix I (around aa 381-384) and that of Helix II (around aa 413-414) are parallel, with α helices aligned in the same direction [52]. Three molecules formed trimerized N throughout the three intermolecular coiled-coil structures.

Two additional alpha-helical structures in the C-terminal region, Helix I and Helix II, were predicted by secondary structure analysis by Kaukinen *et al.* [52]. As shown in Figure 3, Helix I and Helix II contribute to the interaction with other N proteins through the intermolecular coiled-coil structure [52]. With deletion of Helix II from entire N, homotypic interaction did not occur in a yeast two-hybrid assay. On the other hand, N lacking Helix II showed interaction with entire N in the same assay [49]. These results also supported intermolecular interaction between Helix I and Helix II. 

### 2.5. Deduced Structure of Trimerized N Protein

**Figure 4:**
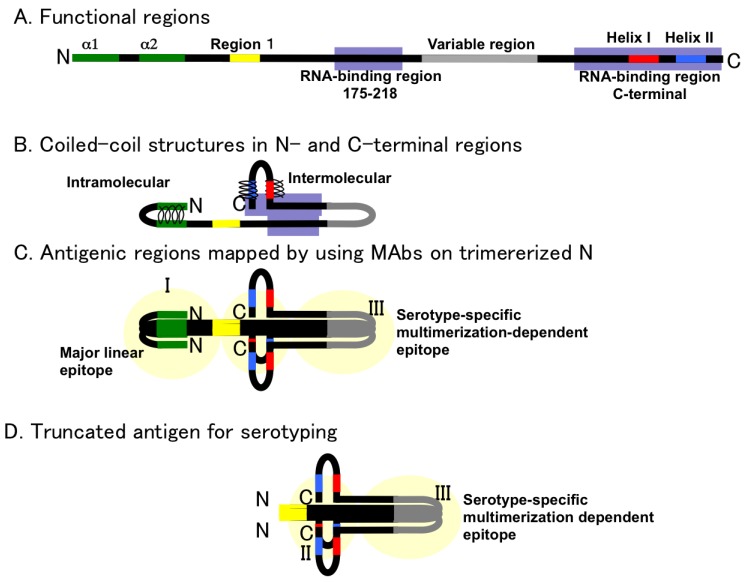
A schema of trimerized hantavirus N. **A.** Functional regions of N were plotted on the primary structure of hantavirus N shown in Figure 1, Figure 2, and Figure 3. **B.** Interactions in the N-terminal and C-terminal part of hantavirus N. **C.** Antigenic regions mapped by poly- and monoclonal antibodies: From the competitive binding assay, two major antigenic regions were found in HTNV N. One was the N terminus (antigenic region I) and the other was the C terminus (antigenic region III). The central region was not a major antigenic site. Serotype-specific epitopes were found in the edge region and as discontinuous epitopes [26]. **D.** Concept of serotyping antigen based on truncation of N antigen. By deletion of group-common and major linear epitopes, serotyping antigens were designed [55].

Mir and coauthors reported that N protein formed a trimer [53]. Based on their observations, a trimerized N protein is presented in Figure 4. In Figure 4A,B, a schema of N protein including coiled-coil interactions in the N and C terminal regions is presented. From epitope mapping of N protein using the competitive binding assay of MAbs, N- and C-terminal regions were in close proximity. Further, a serotype-specific epitope on N protein, which was expected to be located in the central region of N, was found as a projection from the N terminus [26]. According to these observations, an outline of N protein is proposed. In this schema, two RNA-binding regions are in close proximity. These two regions of N protein may cooperatively contribute to RNA binding. In Figure 4C, a schema of trimerized N is presented [54]. Antigenic regions previously designated as I, II, and III [26] were overlaid on the trimerized model. Yoshimatsu *et al.* showed that antigenic regions I and III were immunodominant in actual infection. MAb binding to regions I and III competed with patient sera and infected rodent sera. The antigenicity of region I was reproducible by both *E. coli* and a baculovirus expression system. On the other hand, the antigenicity of region III was reproducible only by the baculovirus expression system [26]. Two HTNV-specific MAb clones, BDO1 [25] and C24B4 [26], were able to bind to N protein expressed by the baculovirus vector. However, recombinant N protein expressed by *E. coli* [26] and the yeast expression system (unpublished observation) showed low binding activity with these HTNV-specific MAbs. To express serotype-specific epitopes among hantavirus N, the selection of an appropriate expression system is important. In Figure 4D, the concept of serotyping antigen based on truncation of N antigen is shown. Morii *et al.* proposed serotyping antigens designed with deletion of group-common and major linear epitopes from N antigen [55]. 

### 2.6. Variety of Trimerization of N Protein Depending on the Viruses and Vectors

Multimerization of N is involved in the antigenicity of N. Most of the serotype-specific epitopes appear to be multimerization-dependent [49]. Authentic hantavirus N proteins in the virion should be multimerized. Native HTNV N proteins in inoculated Vero E6 cells were also detected as multimerized N proteins by competitive sandwich ELISA, as previously described [49]. However, the entire N protein of HTNV expressed in an *E. coli* vector system as described previously was detected as a monomer [26]. On the other hand, recombinant N proteins of SNV, HTNV, and SEOV showed an N-N interaction in yeast and mammalian two-hybrid assays [40,49]. These results indicate that the multimerization of recombinant N protein varied depending on the expression system. On the other hand, recombinant N proteins using the baculovirus expression system were more complicated. As shown in Table 1, all of the recombinant entire N proteins of SNV, ANDV, and LNV were monomeric. Hantavirus N proteins expressed in the baculovirus vector were detected as multimers. Although 155-429 aa of SEOV and DOBV N proteins were detected as multimers, 155-429 aa of HTNV were detected as a monomer. These results indicate that the region of N protein required for multimerization varies among hantaviruses in the baculovirus expression system. They also suggest that the reproducibility of N protein multimerization is dependent on the virus species. Furthermore, as shown in the results for New World hantaviruses except BCCV in Table 1, N-terminal region of the N protein inhibited N-N homotypic interaction.

**Table 1:** Homotypic interactions of recombinant and truncated N proteins.

Region (aa)	HTNV	SEOV	DOBV	THAIV	PUUV	SNV	ANDV	LANV	BCCV	CARV
1-429 (Entire)	M*	M*	M	M	M	S/M*	S	S	M	S
50-429	M*	M*	M	M	M	S	S*	S*	M	ND
100-429	M*	M*	M	ND	M	M	M	M	M	M
155-429	S	M	M	ND	M	S	ND	ND	ND	ND

Multimerization of recombinant and truncated N proteins expressed by the baculovirus vector were examined by competitive binding assay [49,51,56]. M, multimerized N; S, monomeric N; ND, not done. * Furthermore, interactions between entire and/or truncated N proteins were confirmed using the yeast or mammalian two-hybrid assay [49]. BCCV, Black Creek Canal virus.

### 2.7. Association of Cellular Components with N

Interactions among hantavirus N proteins were reported as above. In addition to homotypic interaction of N, cellular proteins associated with N were reported. Although interactions of N protein and small ubiquitin-like modifier-1 (SUMO-1) and its E3 ligase Ubc9 and PIAS were reported, the region responsible for the interaction was the C-terminal region of N [57,58]. Because Nedd4-like ubiquitin ligase E3 was found to be associated with the budding of viral particles from the cellular membrane in the case of Lassa virus, filoviruses, and retroviruses [59,60,61,62], contribution of sumoylation-related enzymes to the budding process of hantavirus virus particles was expected. The binding of N and SUMO-1-related protein seemed to be multimerization-dependent [57]. Mir *et al.* also showed that multimerized N bound to the viral RNA panhandle [53]. These observations suggested that multimerized N might recruit a novel association with a cellular component. On the other hand, Cheng *et al.* reported interaction of N with the ribosomal protein L19 [63,64], and Ramanathan *et al.* showed N traffic in microtubules to the ER-Golgi intermediate compartment (ERGIC) [65,66]. Although regions responsible for N protein binding with L19 or ERGIC remain unclear, it is thought that these interactions participate in viral particle formation. An indirect interaction between N protein and the actin filament or Gn and Gc proteins may also be associated with the assembly of viral particles [67,68,69,70]. 

## 3. Conclusions

Hantavirus N protein is a major antigenic protein. Therefore, it has been important for serological diagnosis. At first glance, it appears simple. However, it has various epitopes such as genus-common, group-common, and serotype-specific epitopes. In addition to the cross-reactivity, both linear and discontinuous epitopes were found from analyses using monoclonal and polyclonal antibodies. Furthermore, the structure that constituted those epitopes has become clear. The N-terminus of N, which was constituted by an antiparallel coiled-coil structure, was found to be immunodominant. On the other hand, the C-terminal half of N, constituted by a parallel intermolecular coiled-coil structure, possessed serotype-specific and multimerization-dependent epitopes. The novel structure of N protein after multimerization might add a novel association with cellular proteins and/or RNA derived from host cells. Finally, reproducibility of epitopes of recombinant antigens is dependent on the expression system and viral species. By understanding the relation of the structure and antigenicity of N protein, a better diagnostic system will be constructed and interpretation of the results of serological tests will be more correct and informative.
